# Comparison of Ultrasonographic Optic Nerve Sheath Diameter Before and After Mannitol Administration in Dogs With Presumed Intracranial Hypertension

**DOI:** 10.1111/vec.70069

**Published:** 2025-11-28

**Authors:** Carlos M. Valerio‐López, Vishal D. Murthy, Sabrina N. Hoehne, John Mattoon, Annie V. Chen

**Affiliations:** ^1^ Department of Veterinary Clinical Sciences, College of Veterinary Medicine Washington State University Pullman Washington USA; ^2^ Department of Surgical and Radiological Sciences, School of Veterinary Medicine University of California, Davis, Davis CA

**Keywords:** biomarker, hyperosmolar therapy, intracranial pressure

## Abstract

**Objective:**

To evaluate ultrasonographic optic nerve sheath diameter (ONSD‐US) as a dynamic biomarker of intracranial hypertension (ICH) following administration of mannitol in patients with clinically suspected ICH.

**Design:**

Prospective observational study over 1 year. Patients were followed for 60 min beyond treatment.

**Setting:**

University teaching hospital.

**Animals:**

Ten prospectively recruited client‐owned dogs with clinically suspected ICH (consecutive sample) and 10 weight‐matched, healthy control dogs.

**Interventions:**

Bilateral transpalpebral ONSD‐US images were collected using a handheld ultrasound probe in dogs with clinically suspected ICH before (*t*_0_) and at 30 min (*t*_30_) and 60 min (*t*_60_) after administration of mannitol therapy (1 g/kg IV). Measurements were collected and evaluated by three observers and compared for agreement. At each time point, a clinical examination was performed, vital parameters were recorded, and neurological scores were assigned using the Modified Glasgow Coma Scale, the Neurological Deficit Score, and the Animal Functional Capacity tools. Bilateral baseline ONSD‐US measurements were also collected from weight‐matched control dogs using the same technique.

**Measurements and Main Results:**

Control dogs had a lower mean (± SD) ONSD‐US (1.6 ± 0.4 mm) than dogs with suspected ICH at baseline (2.0 ± 0.6 mm; *p* = 0.006) on a paired *t*‐test. Among dogs with suspected ICH, compared using a one‐way ANOVA with Tukey's multiple comparisons test, ONSD‐US was decreased from baseline (2.0 ± 0.6 mm) at *t*_30_ (1.8 ± 0.6 mm; *p* = 0.005) and *t*_60_ (1.7 ± 0.5 mm; *p* = 0.003). There was no difference between *t*_30_ and *t*_60_ (*p* = 0.17). Interrater and intrarater reliability were excellent (ICC >0.90). Physiological parameters and neurological scores did not change among the time points assessed, while the Neurological Deficit Score decreased over time (*p* = 0.04; *R* ^2^ = 0.51).

**Conclusions:**

ONSD‐US decreases over time with hyperosmolar therapy and may be a useful noninvasive, dynamic biomarker to identify and monitor ICH and response to therapy.

**Abbreviations**
AFC: Animal Functional Capacity
ICC: intraclass correlation coefficient
ICH: intracranial hypertension
ICP: intracranial pressure
MGCS: Modified Glasgow Coma Scale
NDS: Neurological Deficit Score
ONSD: optic nerve sheath diameter
ONSD‐US: ultrasonographic optic nerve sheath diameter

## 1 Introduction

Intracranial hypertension (ICH) refers to pathologically increased intracranial pressure (ICP) and is often seen in neurologically abnormal patients presenting as medical emergencies with intracranial disease of various etiologies [1, 2]. If left untreated, ICH can impair cerebral perfusion, leading to ischemic injury and neuronal death [2, 3], as well as brain herniation and brainstem compression, which may be fatal [4, 5]. Early identification and emergency treatment of ICH is therefore key to a good outcome [6, 7].

There are limited methods of identifying and monitoring ICH. The Cushing's reflex is one indicator of ICH but only reflects late stages of imminent brain herniation and brainstem compression [8]. The clinical neurological examination can be suggestive of ICH, though a clinical diagnosis of ICH based on neurological examination alone is challenging when patients have abnormal mentation or cranial nerve deficits due to their underlying disease or are administered sedatives or anticonvulsants as part of treatment [4, 9]. Cross‐sectional imaging, such as computed tomography and magnetic resonance imaging, is an alternative that may show intracranial changes consistent with ICH; however, these techniques are expensive, are time consuming, and carry sedation/anesthetic risks to the patient already suffering from neurological impairment, limiting their feasibility, practicality, and safety [10, 11]. The gold standard for measurement of ICP across species is through placement of ICP monitoring catheter systems in the brain parenchyma, ventricles, or subarachnoid space [2, 12]. This is too expensive, invasive, and technically challenging to be practical and available even in most veterinary referral settings [4, 6]. Consequently, there is a need for practical, efficient, and accurate methods to diagnose and monitor ICH in veterinary patients [3, 4, 13, 14]. One method that has shown promise is the use of ultrasound to measure the optic nerve sheath diameter (ONSD).

The optic nerve is surrounded by a sheath formed from an extension of the meninges of the brain, which has a subarachnoid space that is filled with CSF [15]. When pathology in the brain causes ICP to rise, CSF is displaced from the intracranial spaces to the subarachnoid space within the optic nerve sheath, expanding the ONSD measured on ultrasound (ONSD‐US) [4, 16]. In several studies performed in people, bilateral dilation of the ONSD‐US has been established as an early indication of ICH [17, 18, 19, 20].

The ONSD‐US is an easy‐to‐learn and repeatable technique [2, 4, 21], using a modality readily available in many veterinary practice settings. It does not require general anesthesia, is rapid and noninvasive to perform, and is considerably more affordable than advanced cross‐sectional imaging [2, 4, 14]. This can be performed on sedated or obtunded patients that are difficult to accurately assess neurologically [3, 22]. Additionally, expected normal predicted ONSD‐US dimensions have been published in healthy dogs [2].

While ONSD‐US has been shown to be useful in identifying veterinary patients with suspected ICH [3, 4, 22], it has not been specifically studied in other contexts, such as therapeutic monitoring. To be useful for this purpose, ONSD‐US needs to be a dynamic, bidirectional biomarker, sensitive to changes in ICP. We set out to evaluate the utility of ONSD‐US in this context and hypothesized that ONSD‐US in dogs with naturally occurring, clinically suspected ICH will be enlarged compared to healthy dogs of similar weight and will decrease in size following hyperosmolar (mannitol) therapy. If this hypothesis holds true, it would demonstrate the feasibility of ONSD‐US as a practical, dynamic biomarker of ICH. To evaluate this, our aims were to (1) compare baseline ONSD‐US in dogs with clinically suspected ICH prior to hyperosmolar therapy with ONSD‐US in weight‐matched healthy control dogs and (2) compare ONSD‐US at baseline with ONSD‐US at 30 and 60 min following hyperosmolar therapy in dogs with clinically suspected ICH. Additionally, we also (3) recorded and explored changes in clinical variables and neurological functional scores over time following hyperosmolar therapy.

## 2 Materials and Methods

A prospective controlled observational cohort study was performed. Client‐owned adult dogs presenting to the Washington State University Veterinary Teaching Hospital with clinically suspected ICH were enrolled between August 2022 and May 2023. Patients were enrolled with informed client consent, and the study was approved by the Washington State University Institutional Animal Care and Use Committee (Protocol #7036). Clinical suspicion for ICH was defined as compatible neurological examination findings (mentation change, alteration in pupil size, decreased/absent pupillary light reflex, and decreased/absent physiological nystagmus). Not all patients had additional signs of ICH, such as a concurrent Cushing reflex (defined as a Doppler blood pressure >140 mm Hg with concurrent bradycardia) [23, 24] or compatible changes on computed tomography, magnetic resonance imaging, or previously diagnosed brain disease. Healthy, weight‐matched dogs without a previous history of intracranial neurological disease were enrolled in the study as a control group. Patients with primary ocular disease, a history of ocular surgery, or optic neuritis were excluded from both the study and control groups. For the purposes of description, clinical improvement was defined as resolution of one or more neurological abnormalities compared to the previous time point of assessment.

**FIGURE 1:**
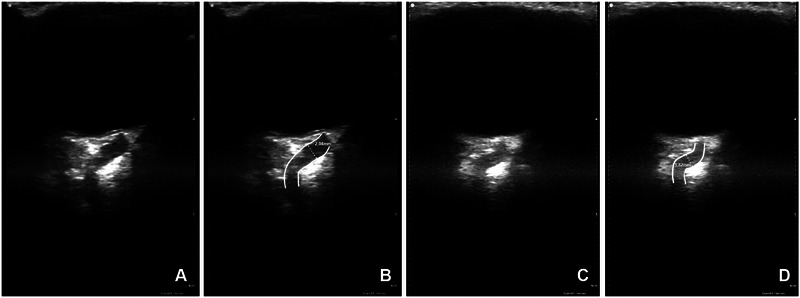
Transpalpebral ocular ultrasonographic image from a 27‐kg, 2‐year‐old dog at baseline (A, B) and 30 min following administration of mannitol (C, D), showing a reduction in optic nerve sheath diameter (ONSD) with treatment. Depth is optimized to visualize the optic nerve in the retrobulbar space. (A) Unmarked image showing visualization of optic nerve sheath at baseline. (B) Same image as A with the optic nerve sheath delineated by white lines and optic nerve sheath diameter (ONSD) measurement overlaid (2.04 mm). (C) Unmarked image of the optic nerve sheath 30 min after mannitol therapy. (D) Same image as C with the optic nerve sheath delineated by white lines and ONSD measurement overlaid (1.82 mm). ONSD in B and D is measured at the point of maximum diameter within 5 mm caudal to the globe.

Enrolled patients from both groups underwent a neurological examination by a neurology resident (C.V.), overseen by two board‐certified neurologists (V.M., A.C.). Optic nerve sheaths were visualized by a trained investigator (C.V.) using a previously described standardized closed eye technique [2] with a handheld, linear‐array veterinary ultrasound transducer 1 and water‐soluble ultrasound transmission gel on unsedated animals. Images were collected in a standardized manner by placing the transducer on the upper eyelid slightly temporal to the globe in the transverse plane (relative to the head), with the probe indicator pointed medially. The transducer was fanned dorsally and ventrally until the optic nerve was visualized along its longitudinal axis. Two sets of images of the optic nerve sheath were collected from each eye and electronically stored, and three sets of measurements were made on each saved image. The ONSD‐US was measured by the first investigator (C.V.) at the time of image acquisition, as previously reported, by placing the measurement calipers on the inner margin of the hyperechoic sheath at the point of maximum diameter, within 5 mm caudal to the optic disc, along the transverse plane (Figure 1) [2, 3]. In the suspected ICH group, baseline ONSD‐US was collected from both eyes, after which mannitol^2^ 1 g/kg IV [25] was administered over 15 min through a 0.2‐micron filter. At 30 min (*t*_30_) and 60 min (*t*_60_) following the completion of mannitol administration, the optic nerve sheath was once again visualized as described above, ONSD‐US was measured, and representative ultrasound images were electronically stored. The control group received a single baseline ultrasound examination, and ONSD‐US was measured with representative images electronically saved.

Patient signalment and clinical variables (mentation, pupillary light reflex, resting pupil size, presence of anisocoria, physiological nystagmus, Doppler blood pressure, and heart rate) were recorded at the time of each ultrasonographic optic nerve sheath examination, and neurological functional scores were assigned using the Modified Glasgow Coma Scale (MGCS) and the Neurological Deficit Score (NDS), as previously described [26, 27, 28]. A previously unvalidated Animal Functional Capacity (AFC) scale was additionally used to score patients [29]. Clinical response to treatment was documented. Diagnoses were made on antemortem magnetic resonance imaging and confirmed on necropsy, where available.

Following collection of two sets of ultrasound images from each eye at each time point per patient in the treatment group, two trained investigators blinded to the treatment status and timing of image collection (A.C., J.M.) independently reviewed saved images in a randomized manner and performed three ONSD‐US measurements on each image as described above, using commercially available imaging software^3^.

### 2.1 Statistical Analysis

Statistical consultation was sought from, and analyses were supported by, the Washington State University Center for Interdisciplinary Statistical Education and Research (CISER). An a priori sample size estimation was performed. Based on a previous feline study [4], which compared healthy controls with patients with ICH (given mean ONSD‐US [healthy] = 1.23 mm; mean ONSD‐US [affected] = 1.65 mm; SD = 0.12 mm), for a power of 80% and *p* = 0.05, two patients per group were estimated to be required to find a statistically significant difference in our study. Further, based on a human study [6] comparing ONSD‐US before and after mannitol administration (mean of paired differences = 0.61 mm; SD = 0.22 mm), for a power of 80% and *p* = 0.05, a total was five patients were estimated to be needed to yield statistically significant differences. While the variability of ONSD‐US is unknown in dogs with ICH, in an effort to account for a suspected higher variability of ONSD‐US due to variation in patient size [2], the sample size was increased to 10 patients in the study population and 10 healthy controls.

All statistical analyses were performed using commercially available statistical software^4^^,^ 5. The measurements collected at the time of ultrasonography by the primary evaluator (C.V.) were used for comparative analyses, and intrarater intraclass correlation coefficients (ICCs) were calculated to assess reliability. The measurements from all three evaluators (C.V., A.C., J.M.) were used to calculate interrater ICC, further testing the reliability of ONSD‐US measurements. Scores of <0.5 (poor reliability), 0.5–0.75 (moderate reliability), 0.75–0.9 (good reliability), and >0.90 (excellent reliability) have been previously reported as guidelines for interpretation of ICC [30]. Interrater ICC estimates for each eye and their 95% confidence intervals were calculated using a single‐rater, absolute‐agreement, two‐way mixed‐effects model [31]. Intrarater ICC estimates for each eye and their 95% confidence intervals were calculated based on a mean rating (*k* = 6), absolute‐agreement, two‐way mixed‐effects model [31].

Data were tested for normality using the Shapiro–Wilk test, which informed the descriptive results and selection of specific statistical comparisons. Statistical significance was set at *p* = 0.05, and calculated *p*‐values were corrected using family‐wise Holm's Sequential Sidak correction [32, 33]. Clinical examination findings are summarized descriptively. Between the dogs with suspected ICH and the healthy weight‐matched controls, age and weight were compared using a Mann–Whitney *U* test or *t*‐test. Sex was compared between groups with a chi‐squared test. To assess whether dogs in the suspected ICH group started off with a greater baseline ONSD‐US averaged for both eyes (OU), dogs were compared to their weight‐matched control counterparts, using a paired *t*‐test.

In the suspected ICH group, mean ONSD‐US measurements for each pair of left eyes (OS) and right eyes (OD) at each time point in each dog were compared using a paired *t*‐test. The mean ONSD‐US OU for each time point per dog was then compared across time using a repeated‐measures one‐way analysis of variance (ANOVA) with Tukey's multiple comparisons test. Heart rate, blood pressure, and MGCS, NDS, and AFC scores were compared across time using a Friedman's test (with Dunn's multiple comparisons test) or repeated‐measures one‐way ANOVA with Tukey's multiple comparisons test as appropriate. Descriptive results of the clinical examination findings are summarized as well.

## 3 Results

### 3.1 Enrolled Study and Control Dogs

A total of 20 dogs were enrolled (10 dogs with suspected ICH, 10 control dogs). A comparison of the age, weight, sex, and ONSD‐US OU in the control and suspected ICH groups is provided in Table 1. The control dogs included two Chihuahua crosses and one each of Cocker Spaniel, Golden Retriever, Labrador Retriever, Miniature Dachshund, Griffon, Greyhound cross, Boston Terrier, and Australian Shepherd cross. The dogs within the suspected ICH group included two Border Collies and one each of Terrier cross, American Eskimo dog, Maltese, Weimaraner, Boxer, Boston Terrier, French Bulldog, and Pug. Of these patients, two had intracranial neoplasia, three had suspected meningoencephalitis of unknown etiology, one had head trauma, one had intracranial hemorrhage, and one had an ischemic infarct. The remaining two dogs had open diagnoses with asymmetrical neurological deficits and seizures, and neither dog survived to receive further diagnostic workup. The top clinical suspicion was for intracranial neoplasia in these cases based on the signalment and history.

**TABLE 1:** Comparison of clinical data parameters and mean optic nerve sheath diameter from both eyes at baseline between dogs with suspected intracranial hypertension and weight‐matched, healthy control dogs.

	Control (*n* = 10)	Suspected ICH (*n* = 10)	*p*‐value
Age [mean ± SD]	4.6 ± 1.9 years	4.1 ± 2.9 years	0.90
Weight [median (range)]	10.9 kg (1.4‐30.0 kg)	11.1 kg (1.1‐31.8 kg)	0.87
Sex [*n* (%)]			0.96
Male	5 (50%)	6 (60%)	
Female	5 (50%)	4 (40%)	
Baseline ONSD‐US OU [mean ± SD]	1.6 ± 0.4 mm	2.0 ± 0.6 mm	0.006^**^

Abbreviations: ICH, intracranial hypertension; ONSD‐US OU, ultrasonographic mean optic nerve sheath diameter from both eyes.

**Significant difference of *p* < 0.01.

### 3.2 Clinical Parameters and Neurological Functional Scores

All dogs in the suspected ICH group showed clinical signs of presumed elevated ICP that warranted hyperosmolar therapy at the time of enrollment. At baseline, a Cushing's reflex was identified in five of 10 dogs, while an altered mentation was seen in all dogs. All dogs showed an abnormal posture, with six of 10 laterally recumbent, three of 10 showing compulsive circling and head pressing, and one of 10 falling to the left. All dogs showed anisocoria, with five of 10 showing unilateral miosis or mydriasis and five of 10 showing bilateral miosis with mild asymmetry. Pupillary light reflexes were reduced in three of 10 dogs and absent in one of 10 dogs. Physiological nystagmus was reduced in four of 10 dogs and absent in one of 10 dogs. At *t*_30_, eight of 10 dogs showed clinical improvement, and at *t*_60_, nine of 10 had improved compared to baseline, though only four of 10 were improved compared to *t*_30_.

The neurological scoring systems’ results are summarized in Table 2. The MGCS showed an overall significant effect of time (*p* = 0.02; Kendall's *W* = 0.48), but no differences were seen between individual time points (baseline to *t*_30_ *p* = 0.943; baseline to *t*_60_ *p* = 0.06; *t*_30_ to *t*_60_ *p* = 0.54). The NDS scores were significantly different overall (*p* = 0.04; *R* ^2^ = 0.51), with differences between baseline and *t*_30_ (*p* = 0.04; Cohen's *d* = 0.93, 95% CI: 0.16–1.66), baseline and *t*_60_ (*p* = 0.02; Cohen's *d* = 1.04, 95% CI: 0.24–1.80), but not *t*_30_ and *t*_60_ (*p* = 0.51; Cohen's *d* = 0.32, 95% CI: −0.3 to 0.86). The AFC scores were not different over time (*p* = 0.47, *R* ^2^ = 0.18).

**TABLE 2:** Comparison of neurological functional scores at baseline (*t*_0_) and 30 min (*t*_30_) and 60 min (*t*_60_) following administration of mannitol in 10 dogs with suspected intracranial hypertension.

	*t*_0_	*t*_30_	*t*_60_
MGCS [median (range)]	14 (6–17)	15 (7–17)	15 (7–18)
NDS (mean ± SD)	169 ± 94	143 ± 89	141 ± 92
AFC (mean ± SD)	7 ± 2	7 ± 2	7 ± 2

Abbreviations: AFC, Animal Functional Capacity scale; MGCS, Modified Glasgow Coma Scale; NDS, Neurological Deficit Score.

The mean heart rate at baseline was 110 ± 50 bpm, at *t*_30_ was 99 ± 33 bpm, and at *t*_60_ was 108 ± 42 bpm. These were not different (*p* = 0.85; *R* ^2^ = 0.04). The Doppler blood pressure at baseline was 148 ± 53 mm Hg, at *t*_30_ was 147 ± 48 mm Hg, and at *t*_60_ was 145 ± 32 mm Hg, which were not different (*p* = 0.96; *R* ^2^ = 0.003).

### 3.3 ONSD‐US

Interrater ICC across all three evaluators for ONSD‐US OS was 0.973 (95% CI: 0.960–0.982) and for ONSD‐US OD was 0.958 (95% CI: 0.930–0.974). Intrarater ICC for the primary evaluator (C.V.) was 0.996 (95% CI: 0.994–0.998) for ONSD OS and 0.995 (95% CI: 0.991–0.997) for ONSD‐US OD. Due to a software glitch, the saved ultrasound images for the baseline ONSD‐US measurements of one dog were lost, precluding repeat measurement by the second and third evaluators. Given the interrater ICC values reported above, this case was deemed highly unlikely to be an outlier, and the saved measurements from the primary evaluator were included in the statistical analyses below. Variability in ONSD‐US measurements within each patient was low, with a mean standard deviation of 0.05 mm at baseline, 0.04 mm at *t*_30_, and 0.04 mm at *t*_60_. The standard deviation across all patients was higher due to variation in patient size and is reported below, along with the mean ONSD‐US at each time point.

Among the dogs with suspected ICH, mean ONSD‐US OS (2.0 ± 0.7 mm) was not different from mean ONSD‐US OD (2.0 ± 0.7 mm; *p* = 0.29; Cohen's *d* = 0.27, 95% CI: 0.10–0.64). Control dogs (1.6 ± 0.4 mm) had a lower mean ONSD‐US OU than dogs with suspected ICH at baseline (2.0 ± 0.6 mm; *p* = 0.006; Cohen's *d* = 1.28, 95% CI: 0.41–2.11) (Table 1). Dogs with suspected ICH showed a reduction in mean ONSD‐US OU following mannitol therapy (overall *p* = 0.001, *R* ^2^ = 0.67). Compared to the baseline mean ONSD‐US OU (2.0 ± 0.6 mm), the mean ONSD‐US OU was smaller at *t*_30_ (1.8 ± 0.6 mm; *p* = 0.005; Cohen's *d* = 1.35, 95% CI: 0.46–2.21) and at *t*_60_ (1.7 ± 0.5 mm; *p* = 0.003; Cohen's *d* = 1.47, 95% CI 0.54–2.37). Mean ONSD‐US OU was not different between *t*_30_ and *t*_60_ (*p* = 0.17; Cohen's *d* = 0.63, 95% CI: −0.07 to 1.30).

## 4 Discussion

Accurate measurement of ICP is challenging in both veterinary and human medicine, and the need for faster, safer, efficient, and cost‐effective measurement techniques has elicited extensive research in both fields. This study is the first to systematically examine the impact of hyperosmolar therapy (mannitol) on ONSD‐US measurements in dogs with suspected ICH. These findings are consistent with Armenise et al. [22], who observed a reduction in unilateral ONSD‐US in two patients treated with mannitol. Our findings also show similar behavior of the ONSD‐US in both eyes, both prior to and following mannitol treatment. Our and others’ data [2, 3, 4, 14] suggest that unilateral ONSD‐US measurement may suffice in certain emergency settings. This may further reduce any delays in initiating treatment and streamline the incorporation of an abbreviated ONSD‐US protocol into routine emergency intake of neurological cases, ideally once ocular disease has been ruled out. On the other hand, it has been suggested by Scrivani et al. [34] that unilateral disease could theoretically alter CSF flow around each optic nerve differently, resulting in different ONSD‐US measurements in each eye. While this is yet to be studied in cases of unilateral intracranial disease, future research and any clinical use should continue to measure ONSD‐US bilaterally to ensure any unilateral changes in ONSD‐US are not missed.

The central finding of our study is the significant reduction in ONSD‐US seen following mannitol administration, which aligns with similar human studies in patients with suspected ICH [6, 12]. Given previous studies correlating direct ICP measurements with ONSD‐US both in veterinary experimental patients and human patients [3, 6], we believe that the ONSD‐US changes observed in our ICH study population likely reflect changes in ICP in response to mannitol administration. These findings provide the first support for the use of ONSD‐US as a biomarker to monitor response to hyperosmolar therapy. However, future research on ONSD‐US measured with concurrent direct ICP monitoring in naturally occurring canine ICH cases may help further support the accuracy and clinical utility of ONSD‐US as a biomarker of ICP in dogs with naturally occurring ICH. Time points of 30 and 60 min post‐mannitol infusion were used as these have been reported to reflect the peak serum osmotic effects of mannitol [25, 35, 36]. Future studies may consider evaluating serial ONSD‐US to identify the times of peak reduction in ONSD‐US, which may or may not correspond to serum osmolarity, given the various other mechanisms of action of mannitol in ICH [35]. The peak effect is likely dose dependent as well. We selected a dose of 1 g/kg of mannitol as it is an accepted and recommended dose in veterinary literature [25]. While there is no definitive evidence for an ideal mannitol dose, studies in humans show a correlation between higher doses of mannitol and greater reductions in ONSD‐US over time [12, 37]. More research is required to evaluate whether the change in ONSD‐US is proportional to the dose of mannitol administered.

Although our study was limited to cases receiving mannitol to minimize potential variability, reductions in ONSD‐US have also been reported after administration of hypertonic saline in humans [38] and would be expected in veterinary patients as well. The authors have noticed a substantial decrease in ONSD‐US following the use of hypertonic saline in patients with ICH, though a more thorough investigation of this was beyond the scope of the current study.

Our results showed that all dogs with suspected ICH had consistently enlarged ONSD‐US compared to their weight‐matched control counterparts. The magnitude of this difference seen in the current study is in line with findings from previous studies [3, 4]. Control cases were matched by weight because body weight has been reported to account for 94.6% of the variation of ONSD‐US [2]. This was the source of the wide standard deviations of the ONSD‐US reported above across patients. The variability in ONSD‐US within patients was considerably lower. A previous study reported predicted normal ranges by body weight, thought to be within 0.2 mm of the true values [2]. It is worth noting that all baseline ONSD‐US values from control dogs in our study fell within the predicted normal ranges for their weights using the equation previously reported [2]. However, these predicted ranges should be interpreted with caution, as they may be exceedingly wide for use as a normal reference range and have not been validated as cutoff values diagnostic of ICH. Results from our suspected ICH population and those from a previous study with experimentally induced ICH monitored with concurrent direct ICP monitoring found that the significantly increased ONSD values often fell within these wide predicted normal ranges, calling into question their validity [3]. In humans, there is less variability in ONSD‐US between patients due to smaller variation in size [6, 12, 39, 40], and ONSD‐US thresholds have been reported to help discriminate between different grades of ICH in varying clinical circumstances [6, 41]. Thus, future research on canine ONSD‐US could examine other potential sources of variation in ONSD‐US and establish cutoff values for the diagnosis of ICH for use in individual patients.

While the methodology used in the present study works well for an individual patient, there is variation between patients, especially those of different sizes, limiting our ability to compare results directly between patients. One method reported to account for interpatient variability involves calculating the ratio of ONSD to the transverse diameter of the eye measured with ultrasound [14, 42]. However, there are conflicting data on the association between ONSD and ocular morphometrics such as the transverse diameter of the eye, and further study of this method is required in dogs [14, 42]. Further, this method has not been validated in canine patients with ICH, unlike the method used in the present report [3]. Lastly, this method utilizes a transcorneal or transscleral approach, requiring the use of topical local anesthetics [14, 42] and increasing the risk of corneal ulcers with repeated examinations, which both add to patient morbidity and result in iatrogenic changes to pupil size, which may cloud the neurological assessment in the context of ICH and intracranial disease.

Excellent reliability was seen within and between investigators measuring ONSD‐US. This is consistent with previous veterinary studies [2, 3, 4, 43], highlights the low variability within patients, and suggests that ONSD‐US measurements may be used reliably within and across patient care team members. Further study into the reliability of the acquisition of serial images by different clinicians over time and across different skill levels is warranted. Variability in ONSD‐US due to patient size limits direct comparisons of the magnitude of change in ONSD‐US between patients. However, within a given patient, this appears to be a reliable biomarker of ICH. While similar excellent reliability is reported in humans, despite less intersubject variation, a recent consensus statement recommended guidelines to standardize the image acquisition and subsequent ONSD‐US measurements in an effort to reduce the variability of the different cutoff values reported [20]. While it is unclear whether the guidelines can be strictly applied to veterinary medicine due to anatomical and functional differences, it is evident that continued research and development of the technique is required to determine which aspects should be standardized in veterinary medicine.

Various clinical scoring tools were examined in the context of ICH in the present study, in addition to the ONSD‐US. These clinical scoring systems have been studied as potential prognostic indicators and biomarkers of disease severity as they account for multiple clinical features at the same time in an ordinal manner [26, 44, 45]. The MGCS is associated with prognosis (survival) in veterinary patients with ICH secondary to head trauma and may improve following mannitol administration [22, 26, 44, 45]. In the present study, the MGCS showed a significant overall difference over time, but there were no differences between individual time points. The present study may simply be underpowered to identify differences in the MGCS between time points, especially with variation in the MGCS between patients. Scoring tools like the MGCS may also simply not be sensitive enough to pick up on subtle but relevant changes in neurological status as patients improve following hyperosmolar therapy. Another scoring tool used in the present study was the NDS [28], which showed a significant change over time, similar to the ONSD‐US, at 30 and 60 min post‐mannitol therapy. This scoring tool is primarily used in cardiopulmonary arrest research [46, 47]. It was included due to its level of detail and a lack of other neurological scoring tools specific to ICH. While this tool is not validated for use in ICH, our findings suggest significant changes in NDS with improvement in ICH, warranting further evaluation of this scale in veterinary patients with ICH. It is important to distinguish the NDS referred to in this study from the Neurodisability Scale recently developed to measure neurological severity in dogs with meningoencephalitis of unknown etiology [48]. Finally, the AFC scale seemed not to be detailed enough to identify significant changes in function as ICH was treated.

The results of this study should be considered in light of some limitations. While efforts were taken to ensure a high level of clinical suspicion for ICH, direct ICP monitoring and cross‐sectional imaging were not possible to confirm the diagnosis of ICH prior to enrollment in all cases. Based on the increasing evidence in human and veterinary medicine that ONSD‐US is a good biomarker of ICP, in the absence of ocular disease, it is expected that our results are a true reflection of ICP variations. However, the changes in ONSD‐US seen in this study would ideally have been confirmed with concurrently measured direct ICP monitoring. The patient population was heterogeneous, with ICH secondary to various brain pathologies and with patients of different sizes. In an effort to minimize the effects of this variation, each dog served as its own control between time points. Serial measurements within an individual patient are more valuable for monitoring and evaluating that patient than comparisons to an absolute normal reference value. There is also a lack of clearly established cutoffs as outlined above. Another limitation was in the acquisition of images. While reliability in measurement was evaluated, all images were collected by a single investigator, and reliability in image acquisition was outside the scope of the current study. Standardized techniques such as the one used in this study should minimize variability in image acquisition. The relatively small sample size limited our ability to draw definitive conclusions regarding variables beyond ONSD‐US, as the study was powered only for this latter variable. Conclusions around neurological severity scores and physiological parameters should be considered in light of this limitation.

Our findings provide further support for the clinical use of ONSD‐US in emergency settings to monitor response to hyperosmolar therapy. This modality may complement neurological examination, both to help in the early identification of ICH as well as to monitor response to treatment. Similar to point‐of‐care thoracic and abdominal ultrasounds, ONSD‐US may be a rapid bedside test of value in neurological patients presenting to the emergency room. It may also have the benefit of identifying ICH earlier than the Cushing reflex, which was seen in only 50% of cases at initial presentation in the present study, while ONSD‐US were already enlarged. This finding is consistent with the notion that the Cushing's reflex is a late‐stage indicator of imminent brain herniation [8]. Future research on the use of ONSD‐US should evaluate whether ONSD‐US may identify ICH prior to the development of different neurological examination findings, correlating back to direct ICP measurements.

## 5 Conclusions

In patients with naturally occurring suspected ICH, ONSD‐US decreases significantly with mannitol therapy, conceivably correlating with reduced ICP. This adds support that ONSD‐US may be useful as a dynamic, noninvasive biomarker of ICP that can be measured as a point‐of‐care test. Applications may include early clinical identification of ICH and monitoring response to mannitol over time. Combining ONSD‐US with neurological examinations may help guide clinical decision‐making in patients with suspected ICH. The continuous use of ONSD‐US in clinical and research settings could add relevant information to develop guidelines and cutoff values that allow the technique to be objectively used in response to dynamic ICP changes.

## Author Contributions

**Carlos M. Valerio‐López**: conceptualization, data curation, formal analysis, funding acquisition, investigation, methodology, validation, writing – original draft, writing – review and editing. **Vishal D. Murthy**: conceptualization, data curation, formal analysis, funding acquisition, project administration, resources, software, supervision, visualization, writing – original draft, writing – review and editing. **Sabrina N. Hoehne**: data curation, formal analysis, software, writing – original draft, writing – review and editing. **John Mattoon**: Formal analysis, investigation, methodology, software, validation, writing – review and editing. **Annie V. Chen**: formal analysis, investigation, methodology, project administration, supervision, validation, writing – review and editing.

## Ethics Statement

The authors confirm that the ethical policies of the Journal, as noted on the Journal's author guidelines page, have been adhered to. This study was approved by the Washington State University Institutional Animal Care and Use Committee (Protocol #7036), and informed client consent was obtained for all subjects enrolled.

## Conflicts of Interest

The authors declare no conflicts of interest.

## Endnotes

1 V‐Scan Air handheld ultrasound; General Electric HealthCare; Chicago, IL.

2 Mannitol 20%, VetOne; MWI Animal Health; Boise, ID.

3 Intelerad; Inteleshare imaging (Formerly AmbraHealth), Raleigh, NC.

4 SPSS v29, SPSS Inc, Chicago, IL.

5 GraphPad Prism v10, GraphPad software LLC, Boston, MA.
